# Incidence and factors associated with rapid blood culture sterilization among patients receiving ertapenem combination therapy for MSSA bacteremia

**DOI:** 10.1128/aac.01303-25

**Published:** 2025-12-23

**Authors:** Sunish Shah, Lloyd G. Clarke, Bonnie A. Falcione, Ryan K. Shields, Brandon J. Smith

**Affiliations:** 1 Antibiotic Management Program, University of Pittsburgh Medical Center 635565, Pittsburgh, Pennsylvania, USA; 2 Department of Pharmacy, University of Pittsburgh Medical Center 15523, Pittsburgh, Pennsylvania, USA; 3 Department of Pharmacy & Therapeutics, University of Pittsburgh School of Pharmacy 15523, Pittsburgh, Pennsylvania, USA; 4 Department of Medicine, University of Pittsburgh 6614, Pittsburgh, Pennsylvania, USA; University of Fribourg, Fribourg, Switzerland

**Keywords:** endocarditis, ertapenem, MSSA

## Abstract

Ertapenem combination therapy has been shown to expedite the time to blood culture sterilization among patients with MSSA bacteremia. The purpose of this study was to define what proportion of patients sterilizes blood cultures within 72 h of ertapenem combination treatment initiation. This was a retrospective, multicenter study of patients who received ertapenem combination therapy for MSSA bacteremia. Patients were considered to have rapid blood culture sterilization if blood cultures were negative within 72 h of ertapenem initiation and delayed blood culture sterilization if blood cultures remained positive after 72 h of ertapenem initiation. Of the 304 patients with MSSA bacteremia who received ertapenem for combination treatment, 197 met the inclusion criteria. The median (IQR) time to ertapenem initiation from the time of blood culture obtainment was 4 (3–5) days, and 47% (93/197) had definitive endocarditis. Overall, 85% (167/197) had rapid blood culture sterilization. Patients who had delayed blood culture sterilization had a trend toward a higher Pitt Bacteremia Score at the time of blood culture obtainment (2 [0–4] vs 1 [0-2], *P* = 0.091) and were more likely to have an oxacillin minimum inhibitory concentration of ≥0.5 µg/mL (67% vs 43%, *P* = 0.015) compared to those with rapid blood culture sterilization. In this large study of patients who received ertapenem combination therapy for MSSA bacteremia, 85% of patients sterilized their blood cultures within 72 h of treatment. These results suggest that a standard 3-day ertapenem course could be a reasonable stewardship strategy for patients initiated on combination therapy for MSSA bacteremia.

## INTRODUCTION

*S. aureus* remains a leading cause of bloodstream infections with a mortality rate that exceeds 25% (1). Multiple studies have shown that rapid blood culture sterilization of MSSA decreases mortality (2, 3). Ertapenem combination therapy with oxacillin, nafcillin, or cefazolin has been shown to expedite the time to blood culture sterilization among patients with MSSA bacteremia (4, 5). However, the optimal duration of the ertapenem combination therapy for MSSA bacteremia remains unclear.

One approach is to continue ertapenem combination treatment until blood cultures sterilize, but this requires 5 days while waiting for the final blood culture results. Considering how quickly blood cultures have been reported to sterilize following ertapenem combination treatment for MSSA and that each day of carbapenem exposure increases the risk of developing a carbapenem-resistant infection by 7%, a standard 3-day duration of ertapenem combination therapy may be considered (4–6). The purpose of this study was to define what proportion of patients sterilize blood cultures within 72 h of ertapenem combination treatment initiation and identify the factors associated with the lack of sterilization despite 72 h of ertapenem combination treatment.

## MATERIALS AND METHODS

This was a retrospective, multicenter study of patients who received ertapenem combination therapy for MSSA bacteremia between March 2020 and June 2025. Patients across nine centers within a single health system were evaluated for inclusion. Patients were excluded if blood cultures were sterilized prior to ertapenem initiation, if they had polymicrobial infection, or if repeat blood cultures were not obtained within 72 h of ertapenem initiation. Patients from our prior analyses were reviewed for inclusion (5, 7). Patients were considered to have rapid blood culture sterilization if blood cultures were negative within 72 h of ertapenem initiation. Patients were considered to have delayed blood culture sterilization if blood cultures remained positive after 72 h of ertapenem initiation.

Patients received ertapenem at a dose of 1 g every 24 h or 500 mg every 24 h if they had a creatinine clearance below 30 mL/min. Definitive endocarditis was defined using the Duke criteria, and the European Society of Cardiology guidelines were used to determine surgical indication (8, 9). Septic shock was defined as a new vasopressor requirement at the time of blood culture obtainment. Nosocomial MSSA bacteremia was defined as bacteremia that occurred after 48 h of hospital admission. Oxacillin minimum inhibitory concentrations (MICs) were determined using automatic susceptibility testing.

Categorical variables were compared by a chi-squared or Fisher’s exact test. Continuous variables were compared using a Wilcoxon rank-sum test. A multivariate logistic regression analysis was performed to assess the predictors of delayed sterilization and included factors with a *P* value < 0.1 in the univariate model. Statistical significance in multivariable and univariate analyses was defined as a *P* value of < 0.05 (two-tailed). Statistical analyses were performed using the software R with the installed commander package (10).

## RESULTS

Of the 304 patients with MSSA bacteremia who received ertapenem for combination treatment, 197 met the inclusion criteria. Among the patients who were excluded, there were 95 patients who had blood cultures sterilized prior to carbapenem initiation, three who had polymicrobial infection, and nine in whom repeat blood cultures were not obtained within 72 h of carbapenem initiation. The median (IQR) time to ertapenem initiation from the time of blood culture obtainment was 4 (3–5) days. The median (IQR) duration of ertapenem treatment was 6 (5–8) days. The median (IQR) age, Charlson Comorbidity Index, and Pitt Bacteremia Score were 59 (44–70), 2 (0–3), and 1 (0–3), respectively. Of the included patients, 28% (55/197) were people who inject drugs, and 47% (93/197) had definitive endocarditis. Among the 93 patients with definitive endocarditis, 57 had surgical indications. Surgical indications for endocarditis included multiple indications (*n* = 19), embolic prevention (*n* = 16), implantable cardiac electronic device (*n* = 8), prosthetic valve (*n* = 5), valve failure (*n* = 5), abscess (*n* = 3), and pseudoaneurysm (*n* = 1). Surgery was not performed despite an indication in 40% (37/93) of patients.

Overall, 85% (167/197) had rapid blood culture sterilization, and 15% (30/197) had delayed blood culture sterilization (Table 1). Three patients in the delayed blood culture sterilization experienced mortality and never had documented sterilized blood cultures. The median (IQR) duration of ertapenem combination therapy was 6 days (4.9–7.3) and 8.5 (6.2–11) in the rapid and delayed sterilization groups, respectively. Patients who had delayed blood culture sterilization had a trend toward a higher Pitt Bacteremia Score at the time of blood culture obtainment (2 [0–4] vs 1 [0–2], *P* = 0.091) and were significantly more likely to have an oxacillin MIC ≥0.5 µg/mL (67% vs 43%, *P* = 0.015) compared to those with rapid blood culture sterilization. Among a subset of patients who received oxacillin or nafcillin with ertapenem (*n* = 94), patients with delayed blood culture sterilization were also more likely to have an oxacillin MIC ≥0.5 µg/mL compared to those with rapid blood culture sterilization (75% [12/16] vs 42% [33/78], *P* = 0.026). However, among the subset of patients who received cefazolin with ertapenem (*n* = 103), patients with delayed blood culture sterilization were not significantly more likely to have an oxacillin MIC ≥0.5 µg/mL (57% [8/14] vs 43% [38/89], *P* = 0.312) compared to those with rapid blood culture sterilization. In the multivariate logistic regression model, an oxacillin MIC ≥0.5 µg/mL was significantly associated with an increased risk of delayed sterilization (OR = 2.6; 95% CI: 1.2–6.6; *P* = 0.021); however, a higher Pitt Bacteremia Score was not significantly associated with an increased risk of delayed sterilization (OR = 1.1; 95% CI: 0.98–1.3; *P* = 0.096).

**TABLE 1:** Univariate analysis of rapid and delayed blood culture sterilization among patients receiving ertapenem combination therapy^*f*^

	Rapid sterilization (*n* = 167)	Delayed sterilization (*n* = 30)	*P* value^*g*^
Patient demographics and underlying diseases			
Age, median (IQR)	58 (43–70)	59 (46–70)	0.855
Female gender, *n* (%)	64 (38)	14 (47)	0.421
CCI, median (IQR)	2 (0–3)	2 (0–4)	0.746
Immunocompromised, *n* (%)	13 (8)	5 (17)	0.161
PWID, *n* (%)	47 (28)	8 (27)	0.869
Diabetes, *n* (%)	56 (34)	12 (40)	0.492
Patient directed discharge, *n* (%)	15 (9)	2 (7)	0.999
Infection characteristics and source control			
Nosocomial bacteremia, *n* (%)	20 (12)	2 (7)	0.539
Pitt Bacteremia score, median (IQR)	1 (0–2)	2 (0–4)	0.091
Septic shock, *n* (%)	36 (22)	11 (37)	0.074
Oxacillin MIC > 1 µg/mL, *n* (%)	11 (7)	4 (13)	0.253
Oxacillin MIC > 0.5 µg/mL, *n* (%)	71 (43)	20 (67)	**0.015**
Body mass index > 30 kg/m^2^, *n* (%)	75 (45)	11 (37)	0.402
Albumin >3 g/dL, *n* (%)	*N* = 157 29 (19)	*N* = 29 2 (7)	0.175
Definitive endocarditis, *n* (%)	77 (46)	16 (53)	0.465
Prosthetic valve endocarditis, *n* (%) Vegetation >1 cm, *n* (%) Septic emboli, *n* (%) Surgery indicated and not performed, *n* (%) Valve surgical management, *n* (%) Time to source control in days, median (IQR)	7/77 (9) 41/77 (53) 62/77 (81) 32/77 (42) 18/77 (23) 14.7 (9.2-22.9)	2/16 (13) 11/16 (69) 13/16 (81) 5/16 (31) 4/16 (25) 24.6 (8.1-40.5)	0.649 0.284 0.999 0.578 0.999 0.932
Spinal infection, n (%) Source control procedure, *n* (%) Time to source control in days, median (IQR)	55 (33) 24/55 (44) 6.5 (3.4–9.3)	13 (43) 6/13 (46) 4.5 (3.5–15.4)	0.261 0.869 0.899
Other source infections, *n* (%)^*a*^ Source control procedure, *n* (%) Time to source control in days, median (IQR)	73 (44) 57/73 (78) 3.2 (1.3–8.2)	12 (40) 11/12 (92) 4.4 (2.4–8.9)	0.705 0.445 0.401
Treatment characteristics			
Empiric antibiotic regimen Vancomycin monotherapy, *n* (%) Vancomycin plus β-lactam, *n* (%) Other, *n* (%)	27 (16) 106 (64) 34 (20)	5 (17) 20 (67) 5 (17)	0.919
Time to empiric treatment in hours, median (IQR)	2.1 (0.02–10)	1.7 (0.23–5)	0.689
Initial targeted regimen selected Oxacillin/nafcillin^*b*^, *n* (%) Cefazolin, n (%)	78 (47) 89 (53)	16 (53) 14 (47)	0.504
Time to targeted treatment in days, median (IQR)^*c*^	2.2 (1.6-2.9)	2 (1.5-3)	0.662
Concomitant anti-*Staphylococcal* antibiotic, *n* (%)^*d*^	20 (12)	3 (10)	0.999
Dose reduced ertapenem	*N* = 167 23 (14)	*N* = 30 5 (17)	0.776
Days of ertapenem, median (IQR)	6 (4.9–7.3)	8.5 (6.2–11)	**< 0.001**
Patient outcomes			
90-day mortality, *n* (%)	34 (20)	11 (37)	0.051
30-day mortality, *n* (%)	26 (16)	5 (17)	0.792
Days to blood culture sterilization from ertapenem initiation, median (IQR)^*e*^	*N* = 167 0.9 (0.6-1.8)	*N* = 27 4.4 (3.9–5.6)	**< 0.001**
Length of hospital stay from time of blood culture, median (IQR)	17 (11–27)	20 (13–31)	0.305

^*a*^ Diabetic foot infections (*n* = 4), endophthalmitis (*n *= 2), central venous catheter (*n* = 10), multiple non-endovascular or non-spinal sources (*n* = 9); other sources (*n* = 10); prosthetic joint/septic arthritis (*n* = 26); prostate abscess (*n* = 3); pulmonary (*n* = 8); skin/soft tissue infection (*n* = 7); sternal infection (*n* = 5).

^*b*^ There were 91 patients who received oxacillin and 3 patients who received nafcillin.

^*c*^ Rapid molecular testing of *S. aureus* without the presence of mecA was used in 22% of (43/197) cases.

^*d*^ Other agents received included rifampin (*n* = 17), aminoglycosides (*n* = 1), daptomycin (*n* = 1), or rifampin plus gentamicin (*n* = 4).

^*e*^ Three patients experienced mortality and failed to sterilize blood cultures.

^*f*^ CCI, Charlson comorbidity index; PWID, people who inject drugs.

^*g*^ Bold values indicate *P* < 0.05.

Overall, the 90-day mortality rate was 23% (45/197). Numerically, 90-day mortality in the delayed sterilization group was higher compared with the rapid sterilization group (37% vs 21%, *P* = 0.057). Ertapenem combination therapy was well tolerated in all patients except for one patient who developed a seizure that required ertapenem discontinuation. This patient had a history of seizures and had ertapenem discontinued after 3 doses at 1 g every 24 h.

## DISCUSSION

This is the largest study to date of patients who received ertapenem combination therapy for MSSA bacteremia. We found that 85% of patients sterilized their blood cultures within 72 h of treatment, despite nearly half of the cohort having definitive endocarditis. Therefore, these results suggest that a standard 3-day ertapenem course could be a reasonable stewardship strategy for patients initiated on ertapenem combination therapy for MSSA bacteremia. This approach allows for rapid sterilization of blood cultures while limiting carbapenem exposure.

Our prior experience with carbapenem combination therapy demonstrated a faster time to blood culture sterilization by approximately 25 h compared with matched control patients who remained on standard treatment (5). However, other clinical advantages, such as lower mortality, shorter lengths of stay, or decreased rates of recurrence, were not demonstrated with this combination, which would support discontinuation of the carbapenem following blood culture sterilization. A similar approach has been studied for MRSA bacteremia in which de-escalation of combination therapy with ceftaroline yielded comparable outcomes to those who remained on ceftaroline combination therapy for the entire course (11). Nevertheless, the optimal duration of ertapenem combination therapy for MSSA bacteremia remains unclear. Notably, the ongoing CERT SNAP clinical trial is estimated to randomize 60 patients to receive cefazolin with placebo or ertapenem combination therapy for 5 days (ClinicalTrials.gov ID NCT04886284). In this real-world study, the median duration of ertapenem combination therapy was 6 days, with only 12% (23/197) of patients receiving 3 days of ertapenem or less. This emphasizes an opportunity to decrease the duration of ertapenem therapy if a standard 3-day approach were implemented.

Patients with delayed clearance had a numerical trend toward a higher Pitt bacteremia score and septic shock. A higher inoculum of infection or suboptimal pharmacokinetics in critical illness may contribute to delayed blood culture sterilization, and in this population, further research utilizing therapeutic drug monitoring is required (12). An association was found between elevated oxacillin MICs ≥ 0.5 µg/mL and delayed blood culture sterilization. Although patients with elevated oxacillin MICs may also represent a patient population where ertapenem combination therapy requires further study, it is recognized that oxacillin MICs were not performed by standard broth microdilution. Nevertheless, observational data in patients with MSSA bacteremia managed with cefazolin or anti-staphylococcal penicillins have not shown oxacillin MICs to impact treatment outcomes (13). Furthermore, the association between oxacillin MICs ≥ 0.5 µg/mL and delayed blood culture sterilization was not significant in the subset of patients who had received cefazolin with ertapenem. Preliminary results from the SNAP trial found cefazolin to be non-inferior to flucloxacillin for 90-day mortality in patients being treated for MSSA bacteremia, and fewer adverse effects were noted among cefazolin users (ClinicalTrials.gov ID NCT05137119). Therefore, cefazolin may be considered standard in the treatment of MSSA bacteremia.

Although the strength of this study lies in its large sample size, several limitations are recognized. First, the observational design of this study did not allow for consistency with blood culture obtainment. Although we attempted to control for this by only including patients who had blood cultures obtained within 72 h of ertapenem initiation, it is possible that many patients may require even less than 72 h of carbapenem therapy to sterilize blood cultures. For instance, a case series that evaluated ertapenem combination therapy for persistent MSSA bacteremia and endocarditis found that 88% (8/9) of patients who had daily blood cultures obtained experienced blood culture sterilization within 24 h (4). Second, it is recognized that 95 patients were excluded since they had sterilized blood cultures with antimicrobials before ertapenem was ever initiated. Therefore, it is expected that many patients within our included cohort would have eventually sterilized blood cultures without ertapenem combination therapy. Since a large portion of patients had sterilized blood cultures prior to ertapenem initiation, future studies may focus on strategies to avoid the initiation of ertapenem therapy altogether. Finally, only 30 patients in this exploratory analysis had delayed blood culture sterilization, which limited our ability to identify risk factors associated with the phenomenon. For instance, after propensity score matching, we recently found that among patients with persistent MSSA bacteremia who were treated with ertapenem combination therapy, patients with hypoalbuminemia had a significantly longer time to negative blood cultures (7). However, in the current study, the rate of hypoalbuminemia was numerically, but not statistically, higher among those with delayed blood culture sterilization.

### Conclusion

In this large study of patients who received ertapenem combination therapy for MSSA bacteremia, 85% of patients sterilized their blood cultures within 72 h of treatment. These results suggest a standard 3-day ertapenem course could be a reasonable stewardship strategy for patients initiated on combination therapy for MSSA bacteremia.
